# Illness-Death Model in Chronic Disease Epidemiology: Characteristics of a Related, Differential Equation and an Inverse Problem

**DOI:** 10.1155/2018/5091096

**Published:** 2018-09-12

**Authors:** Ralph Brinks

**Affiliations:** ^1^Hiller Research Unit for Rheumatology, University Hospital Duesseldorf, Duesseldorf, Germany; ^2^Institute for Biometry and Epidemiology, German Diabetes Center, Duesseldorf, Germany

## Abstract

Chronic diseases impose a huge burden for mankind. Recently, a mathematical relation between the incidence and prevalence of a chronic disease in terms of a differential equation has been described. In this article, we study the characteristics of this differential equation. Furthermore, we prove the ill-posedness of a related inverse problem arising in chronic disease epidemiology. An example application for the inverse problem about type 1 diabetes in German women aged up to 35 years is given.

## 1. Introduction

**Figure 1:**
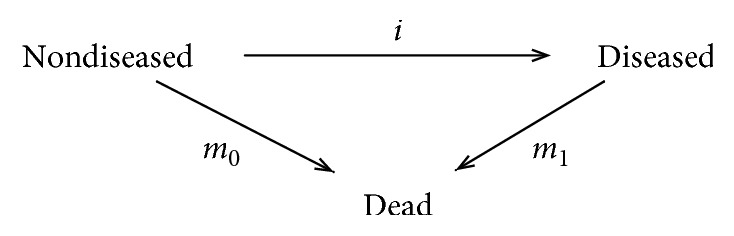
Illness-death model of a chronic disease. The rates *i*, *m*_0_, and *m*_1_ describe the transitions between the states.

Chronic diseases impose an enormous burden for mankind. It has been estimated that 71% of the 56 million global deaths in 2015 were attributable to noncommunicable diseases with an upward trend in the past decades [1]. Leading causes of death in 2015 were ischemic heart disease and cerebrovascular disease. Both are irreversible, i.e., chronic, conditions. Modelling of chronic conditions is often accomplished by compartment models like the illness-death model shown in Figure 1.

The illness-death model goes back at least until the 1950s [2] and consists of the three states: *nondiseased*, *diseased*, and *dead* [3, 4].

The prevalence of a chronic disease can be related to the transition rates in the illness-death model by a scalar partial differential equation [5]. Using the method of characteristics [6], this partial differential equation can be reduced to an ordinary differential equation (ODE), which pictures the temporal change of the prevalence along the characteristic lines. Thus, one time variable and a scalar ODE are sufficient to describe the change of the prevalence as a function of the transition rates in the illness-death model. Until now, a rigorous mathematical treatment of the ODE is missing.

The ODE has an important epidemiological application. Given the mortality rates (*m*_0_  and  *m*_1_ in Figure 1) and the age-specific prevalence, the ODE may be used to derive the incidence rate (*i*). Estimating the incidence rate of a disease is an important epidemiological problem [3, 7, 8]. This task can be interpreted as an inverse problem, which is often examined with respect to ill- or well-posedness. A well-posed inverse problem in the sense of Hadamard means that a solution for the inverse problem exists and that the solution is unique and continuous [9]. In this article, the ill-posedness of the inverse problem is proven, and an example application from the field of diabetes is given.

This article is organized as follows: Section 2 reviews the derivation of the ODE. Then, some properties of the ODE and its solution are examined. In Section 3, the inverse problem is introduced and the ill-posedness of the inverse problem is proven. To demonstrate the importance and applicability of the theory, an example for the inverse problem is given. Finally, in Section 4, the results and their consequences are discussed.

## 2. Derivation and Properties of the ODE

A popular framework for studying relations between prevalence and incidence of a chronic disease is the illness-death model shown in Figure 1. People in the population under consideration can contract the disease at the incidence rate *i*, and they can die either with the disease at the age-specific mortality rate *m*_1_ or without the disease at the mortality rate *m*_0_. The numbers of individuals in the *nondiseased* state and in the *diseased* state are denoted by *S* (susceptibles) and *C* (cases). Both numbers, *S* and *C*, are assumed to be sufficiently large, such that they can be considered as continuously differentiable functions. As described in Introduction, one time variable, the age *a*, *a* ≥ *a*_0_ ≥ 0, is sufficient to describe the temporal evolution of the population in the illness-death model.

We additionally assume that the population is closed, i.e., there is no migration. Furthermore, the age-specific functions *i*, *m*_0_,  and  *m*_1_ are nonnegative and differentiable in [*a*_0_, *ω*] for some *ω* ∈ *ℝ* ∪ {*∞*} with *ω* > *a*_0_. Henceforth, *ω* is considered as age when all members (diseased and nondiseased) of the population are deceased. Then, the system of ODEs given by (1) and (2) describes the change rates of the numbers *S* and *C* of nondiseased and diseased individuals, respectively:

(1) $$\begin{array}{c} \frac{dS}{da}=-\left(i+m_{0}\right)S, \end{array}$$

(2) $$\begin{array}{c} \frac{dC}{da}=iS-m_{1}C. \end{array}$$

The system (1) and (2) is linear and of first order. Due to the simple structure of the ODEs for a given age-specific incidence *i* and mortality rates *m*_0_ and *m*_1_, the analytical solution of the corresponding initial value problem with initial conditions *S*(*a*_0_)=*S*_0_ ≥ 0  and  *C*(*a*_0_)=*C*_0_ ≥ 0 with *S*_0_+*C*_0_ > 0 can be calculated easily:

(3) $$\begin{array}{c} S\left(a\right)=S_{0}\exp\;\left(-\int_{a_{0}}^{a}i\left(\tau \right)+m_{0}\left(\tau \right)\;\;d\tau \right), \end{array}$$

(4) $$\begin{array}{c} C\left(a\right)=\exp\;\left(-\int_{a_{0}}^{a}m_{1}\left(\tau \right)\;\;d\tau \right)\left\{C_{0}+\int_{a_{0}}^{a}i\left(\tau \right)S\left(\tau \right)\;\exp\;\left(\int_{a_{0}}^{\tau }m_{1}\left(t\right)\;\;dt\right)\;\;d\tau \right\}. \end{array}$$

Obviously, from *S*_0_+*C*_0_ > 0, it follows that *S*(*a*) ≥ 0, *C*(*a*) ≥ 0, and *S*(*a*)+*C*(*a*) ≥ 0 for all *a* ∈ [*a*_0_, *ω*].

Sometimes, it is important to consider the number *N*=*N*(*a*) of persons aged *a*, *a* ≥ *a*_0_, who are alive, i.e., *N*(*a*)=*S*(*a*)+*C*(*a*). For *N*, we have the following initial value problem:

(5) $$\begin{array}{c} \frac{dN}{da}=-m_{0}S-m_{1}C, \end{array}$$

with initial condition *N*(*a*_0_)=*S*(*a*_0_)+*C*(*a*_0_) > 0. From Equation (5), we find *dN*/*da*=−*m*_1_*N*+(*m*_1_ − *m*_0_)*S*=−*m*_1_*N*+Δ*mS*, where Δ*m*=*m*_1_ − *m*_0_. This yields

(6) $$\begin{array}{c} N\left(a\right)=\exp\left(-\int_{a_{0}}^{a}m_{1}\left(\tau \right)\;d\tau \right)\left\{N_{0}+\int_{a_{0}}^{a}\Delta m\left(\tau \right)S\left(\tau \right)\;\exp\;\left(\int_{a_{0}}^{\tau }m_{1}\left(t\right)\;\;dt\right)\;d\tau \right\}. \end{array}$$

For chronic diseases, it is reasonable to assume Δ*m* ≥ 0. Then, from Equation (6), we see that *N*(*a*) ≥ 0 for all *a* ∈ [*a*_0_, *ω*]. We may conclude

**Theorem 1 .**

For nonnegative and differentiable functions *i*, *m*_0_, *and*  *m*_1_ : [*a*_0_, *ω*]⟶*ℝ*, with Δ*m*(*a*)=*m*_1_(*a*) − *m*_0_(*a*) ≥ 0 for all *a* ∈ [*a*_0_, *ω*], the system of ODEs given by equations (1) and (2) with initial conditions *S*(*a*_0_)=*S*_0_ ≥ 0, *C*(*a*_0_)=*C*_0_ ≥ 0, *and* *S*(*a*_0_) +*C*(*a*_0_) > 0 has a unique solution *S* *and* *C* with *N*(*a*)=*S*(*a*)+*C*(*a*) ≥ 0 for all *a* ∈ [*a*_0_, *ω*].

From *N*(*a*) ≥ 0 for all *a* ∈ [*a*_0_, *ω*], we can infer

*Definition 1*. With the assumptions of Theorem 1, the function *p* : [*a*_0_, *ω*]⟶*ℝ* with

(7) $$\begin{array}{c} p\left(a\right)=\left\{\begin{array}{cc} \frac{C\left(a\right)}{N\left(a\right)} & \text{for}\;\;N\left(a\right)>0,\\ 0 & \text{for}\;\;N\left(a\right)=0, \end{array}\right. \end{array}$$

is well defined for all *a* ∈ [*a*_0_, *ω*]. The function *p* in Equation (7) is called the *age-specific prevalence*.

Next, we show that, with the assumptions of Theorem 1, the age-specific prevalence is epidemiologically meaningful.

**Theorem 2 .**

With the assumptions of Theorem 1, the age-specific prevalence *p* : [*a*_0_, *ω*]⟶*ℝ* defined by Equation (7) is bounded with

(8) $$\begin{array}{c} p\left(a\right)\in \left[0,1\right]\;for\;all\;\;a\in \left[a_{0},\omega \right]. \end{array}$$

*Proof*. Let *a* ∈ [*a*_0_, *ω*]. If *C*(*a*)=0, then it is *p*(*a*)=0. For *C*(*a*) ≠ 0, we have *p*(*a*)=1/(1+*x*) with *x*=(*S*(*a*)/*C*(*a*)) ≥ 0. This proves *p*(*a*) ≤ 1. From Equation (4), we see that *C*(*a*) ≥ 0, which implies *p*(*a*) ≥ 0.

Interestingly, the two-dimensional system (1) and (2) can be reduced to a scalar ODE.

**Theorem 3 .**

Let *i*, *m*_0_, *and*  *m*_1_ : [*a*_0_, *ω*]⟶*ℝ* be nonnegative and differentiable functions with Δ*m*(*a*)=*m*_1_(*a*) − *m*_0_(*a*) ≥ 0 for all *a* ∈ [*a*_0_, *ω*]. Let (*S*, *C*) : [*a*_0_, *ω*]⟶*ℝ*^2^ be the unique solution of the system (1) *and* (2) with initial conditions *S*(*a*_0_)=*S*_0_ ≥ 0, *C*(*a*_0_)=*C*_0_ ≥ 0, *and*  *S*(*a*_0_)+*C*(*a*_0_) > 0. Then, *p*=*C*/(*S*+*C*) is differentiable and the unique solution of the ODE is

(9) $$\begin{array}{c} \frac{dp}{da}=\left(1-p\right)\;\left\{i-p\left(m_{1}-m_{0}\right)\right\}, \end{array}$$

with initial condition *p*(*a*_0_)=*C*_0_/(*C*_0_+*S*_0_).

*Proof*. Essentially, this follows from the quotient rule applied to *p*=*C*/(*S*+*C*) and inserting Equations (1) and (2).

**Figure 2:**
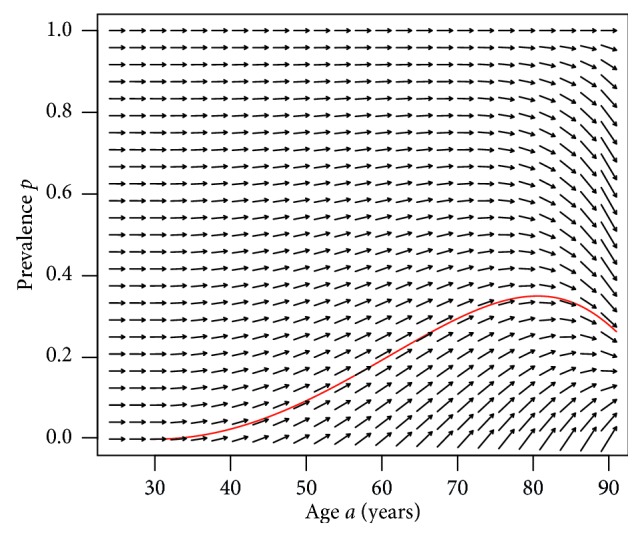
Slope field and solution of an associated initial value problem (red) in Example 1.

*Example 1*.  Figure 2 shows the slope field of an exemplary ODE (9) with the incidence rate chosen to be *i*(*a*)=(max(0, *a* − 30))/2000. The mortality rates *m*_0_ and *m*_1_ are assumed to be of Gompertz type:

(10) $$\begin{array}{c} m_{j}=\exp\;\left(\beta_{0,j}+\beta_{1,j}a\right),\;j=0,\;\;1, \end{array}$$

with the coefficients *β*_*k*,*j*_, *k*, *j*=0, 1, as shown in Table 1.

The slope field in Figure 2 shows the solution of an associated initial value problem with initial condition *p*(30)=0 (red line). The existence of a (local) maximum of the age-specific prevalence (here at an age of about 80 years) is typical for many chronic diseases, e.g., dementia [10], diabetes [11], or rheumatic diseases [12].

The ODE (9) is of Riccati type [13]. In epidemiological contexts, the mortality *m*_0_ of the nondiseased people is usually unknown. Frequently, for a population under consideration, the overall mortality rate *m* (general mortality) is known from vital statistics. The mortality rate *m* is a convex combination of the mortality rate *m*_0_ of the nondiseased people and the mortality rate *m*_1_ of the diseased people:

(11) $$\begin{array}{c} m\left(a\right)=p\left(a\right)m_{1}\left(a\right)+\left\{1-p\left(a\right)\right\}{\;m}_{0}\left(a\right)\\ =m_{0}\left(a\right)\;\left\{p\left(a\right)\left(R\left(a\right)-1\right)+1\right\}, \end{array}$$

where *R*(*a*) is the relative risk, *R*=*m*_1_/*m*_0_.

Apart from the incidence rate *i*, two pieces of information about the mortality are necessary to solve the ODE (9). For instance, *m* and *R* are sufficient to determine the right-hand side of Equation (9). Depending on the type of information about the mortality (*m*_0_, *m*_1_, *m*, or *R*) from the epidemiological context, the ODE (9) changes its type, which is important when solving the ODE. The possible combinations are shown in Table 2. In case the ODE is linear, an easy analytical solution exists. If the ODE is of Riccati or Abelian type [13], a general analytical solution does not exist. An extensive monograph about Riccati equations is in [14].

The fractions (*R* − 1)/*R* and (*p*(*R* − 1))/(*p*(*R* − 1)+1) in the last two rows of Table 2 are well-known quantities in epidemiology. These are the *exposition attributable fraction* (EAF) and the *population attributable fraction* (PAF), respectively [15].

*Remark 1*. For the special case that *m*_0_(*a*)=*m*_1_(*a*) for all *a* ∈ [*a*_0_, *ω*]—this case is called *nondifferential mortality*—the solution of Equation (9) with initial condition *p*(*a*_0_)=*p*_0_ ∈ [0, 1] is

(12) $$\begin{array}{c} p\left(a\right)=1-\left(1-p_{0}\right)\;\exp\;\left(-\int_{a_{0}}^{a}i\left(\tau \right)\;d\tau \right). \end{array}$$

Starting from the system of ODEs (1) and (2), we have deduced the scalar ODE (9), which is well defined and epidemiologically meaningful. At the end of this section, we prove that the reverse way—from ODE (9) to the system (1) and (2)—is also possible. Before we can prove this, we need a slightly modified version of ODE (5).

**Lemma 1 .**

With the general mortality m defined in Equation (11), the ODE (5) can be reformulated into

(13) $$\begin{array}{c} \frac{dN}{da}=-mN, \end{array}$$

with initial condition *N*(*a*_0_)=*N*_0_ > 0.

Then, from calculus, we can deduce the following correspondence between the system (1) and (2) and the two ODEs (9) and (13):

**Theorem 4 .**

(correspondence theorem). Let *i*, *m*_0_, *and*  *m*_1_ : [*a*_0_, *ω*]  ↦  *ℝ* be nonnegative differentiable functions.

- If *S*(*a*)and *C*(*a*) are solutions of (1) and (2) with *S*(*a*_0_)=*S*_0_ ≥ 0, *C*(*a*_0_)=*C*_0_ ≥ 0, and  *S*_0_+*C*_0_ > 0, then *p*(*a*)≔*C*(*a*)/(*S*(*a*)+*C*(*a*)) and *N*(*a*)≔*C*(*a*) +*S*(*a*) are solutions of (9) and (13) with *p*(*a*_0_)=*C*_0_/(*S*_0_+*C*_0_) and *N*(*a*_0_)=*S*_0_+*C*_0_. The general mortality *m* ∈ *C*^0^([*a*_0_, *ω*]) in Equation (13) is defined by (11).
- If *p*(*a*)and *N*(*a*) are solutions of (9) and (13) with *m*=*pm*_1_+(1 − *p*)*m*_0_ and initial conditions *p*(*a*_0_)=*p*_0_ ∈ [0, 1] and *N*(*a*_0_)=*N*_0_ > 0, then *S*(*a*)≔{1 − *p*(*a*)}*N*(*a*) and *C*(*a*)≔*p*(*a*)*N*(*a*) are solutions of (1) and (2) with initial conditions *S*(*a*_0_)=(1 − *p*_0_)*N*_0_ and *C*(*a*_0_)=*p*_0_*N*_0_.

*Proof*. Part (a) follows largely from Theorem 3 and the fact that (*dN*/*da*)=(*dS*/*da*)+(*dC*/*da*). For part (b), we have to apply the product rule to *S*=(1 − *p*)*N* and *C*=*pN*. An easy calculation yields the results about the initial conditions.

*Remark 2*. In [14], it has been shown that any solution *w* of a Riccati ODE corresponds to a solution *y*=(*u*, *v*) of a two-dimensional system of linear ODEs with *w*=*u*/*v*. It can be shown that the functions *C* and *N* have the roles of *u* and *v*, respectively. Here, Theorem 4 has been tailored to the epidemiological context.

## 3. The Inverse Problem

A key application for the ODE (9) is the derivation of the age-specific incidence rate *i* from the age-specific prevalence *p* if the mortalities (or any equivalent information in the first column of Table 2) are known. In epidemiology, incidences rates are typically surveyed in follow-up studies, which may be lengthy and costly. However, Equation (9) allows a new way of estimating the incidence *i*. Besides mortality information, the age-specific prevalence (*p*) has to be known, which can be obtained from relatively cheap cross-sectional studies. An example about type 1 diabetes is shown below.

In such an application with given mortalities, we conclude from an effect (the prevalence) the underlying cause (the incidence), which can be interpreted as an *inverse problem* [16]. The inverse problem is opposed to the *direct problem* of inferring from the incidence (i.e., the cause) the prevalence (the effect) by ODE (9).

### 3.1. Ill-Posedness of the Inverse Problem

We show that the inverse problem is ill-posed in the sense of Hadamard [9]. Let the mortalities *m*_0_ and *m*_1_ be continuous and nonnegative (in this section, *C*^*k*^([*a*_0_, *ω*]) denotes the set of all *k*-times continuously differentiable real-valued functions, *k*=0, 1). For *p*_0_ ∈ [0, 1], define the operator *℘* : *C*^0^([*a*_0_, *ω*])⟶*C*^1^([*a*_0_, *ω*]), *i*  ↦  *p*, such that *p*(*a*_0_)=*p*_0_ and *p* is the solution of (9). To show that the inverse problem is ill-posed, we prove that *℘*^−1^ : *p*  ↦  ((*dp*/*da*)/(1 − *p*)) +*m* − *m*_0_ is discontinuous. It is sufficient to show this for the special case of nondifferential mortality (*m*=*m*_0_). Let *C*^*k*^([*a*_0_, *ω*]), *k*=0, 1, be equipped with the *C*^*k*^ norm ||·||. Choose *p* ∈ *C*^1^([*a*_0_, *ω*]), *ε* > 0, and define *p*_*ε*,*n*_(*a*)≔*p*(*a*)+*ε*sin(*na*). Then, it is *p* − *p*_*ε*,*n*_ ≤ *ε* and

(14) $$\begin{array}{c} \left\|{℘}^{-1}\left(p\right)-{℘}^{-1}\left(p_{\varepsilon ,n}\right)\right\|=\left|\left|\frac{dp}{da}\frac{1}{1-p}-\frac{dp_{\varepsilon ,n}}{da}\frac{1}{1-p_{\varepsilon ,n}}\right|\right|\\ =\left\|\frac{\left(dp/da\right)\varepsilon \;\;\sin\left(n\cdot \right)+\varepsilon n\;\;\cos\left(n\cdot \right)\left(1-p\right)}{\left(1-p\right)\left(1-p-\varepsilon \;\;\sin\left(n\cdot \right)\right)}\right\|. \end{array}$$

For *ε*_*n*_≔*n*^−1/2^ and *p*(*a*) ≠ 1, the term *ε*_*n*_*n*  cos(*na*)(1 − *p*(*a*)) is unbounded as *n*⟶*∞*, which implies that *℘*^−1^ is discontinuous and the inverse problem is ill-posed.

### 3.2. Example: Incidence of Type 1 Diabetes

**Figure 3:**
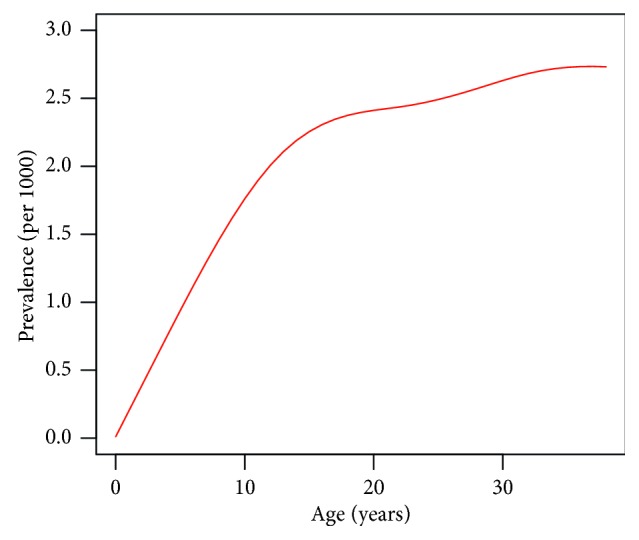
Age-specific prevalence of type 1 diabetes in German women aged 35 and below in 2010.

In this section, the inverse problem is solved in the context of type 1 diabetes. Type 1 diabetes is a chronic condition that mostly arises in the early decades of life.  Figure 3 shows the age-specific prevalence (*p*) of type 1 diabetes in German women aged up to 35 years in the year 2010. Diagnoses stem from health insurance claims of 65 million people. Details about the data collection are given in [11]. The age-specific prevalence is steeply increasing from birth to the age of about 15 years. At age 20, a first plateau is reached, and then a second increase up to age 30 can be observed. At age 35 a second plateau is reached.

To estimate the age-specific incidence (*i*), we use the following equation:

(15) $$\begin{array}{c} i=\frac{dp/da}{1-p}+m\frac{p\left(R-1\right)}{p\left(R-1\right)+1}, \end{array}$$

where *m* is the general mortality for the year 2010 and *R* is the relative mortality risk. The general mortality is publicly available from the Federal Statistical Office of Germany. The population-wide relative mortality *R* is unknown for Germany. Thus, we consider two *extreme* scenarios: *R*=0.5 and *R*=5, and one realistic scenario: *R*=1. Choosing the two extreme scenarios is done because the true (but unknown) age-specific incidence rate will be located between those rates derived from the two extreme scenarios (*sandwich principle*). The scenario *R*=0.5 assumes that women with type 1 diabetes undergo half the mortality rate of women without diabetes, which means that type 1 diabetes is a protective factor against death. The second scenario *R*=5 considers type 1 diabetes to be a strong risk factor for death and that women with type 1 diabetes undergo a fivefold mortality rate than those women without type 1 diabetes. Both scenarios are unrealistic, and the true relative mortality risk is certainly somewhere between these two extreme scenarios. Empirical data from countries with comparable health care systems indicate that *R* is approximately 3 [17, 18]. In case of patients with severe late complications, *R* reaches a value of about 4 [19].

**Figure 4:**
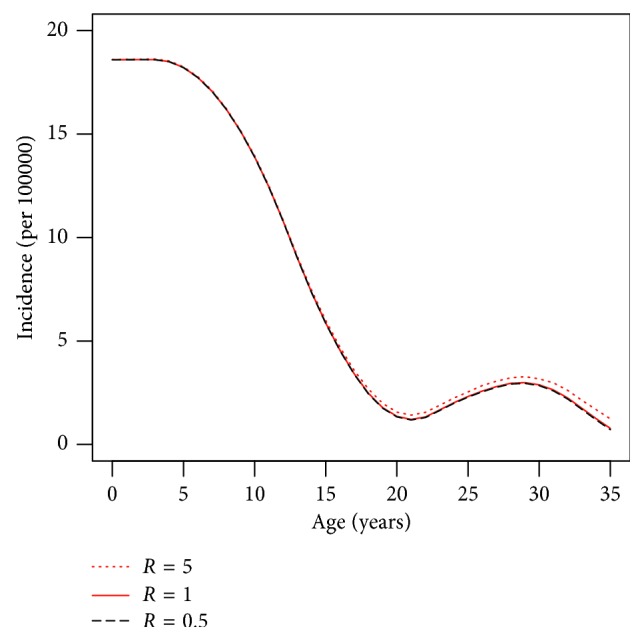
Age-specific incidence of type 1 diabetes in German women in 2010. The different scenarios in the relative mortality *R* are indicated by different line types.

Figure 4 shows the age-specific incidence rate of type 1 diabetes in German women in 2010. The different scenarios of the relative mortality (*R*=0.5, 1,  and  5) are indicated by different line types. There is no visual difference between all of the scenarios until age 20. For age 20+, the scenarios *R*=1 and *R*=0.5 are virtually indistinguishable. The scenario *R*=5 leads to a slightly elevated incidence rate compared to the scenario *R*=1. At age 35, the difference is 0.43 per 100000.

All three scenarios unveil that the age-specific incidence of type 1 diabetes decreases from birth to the age of 20 years. Then, a second peak of the age-specific incidence rate occurs at the age of about 28 years.

## 4. Discussion

By extending the framework in [4] for studying the relation between prevalence and incidence, it had been found that prevalence, incidence, and mortality are linked by a one-dimensional ODE [20]. In this article, it has been shown that the solutions of this ODE are epidemiologically meaningful. Depending on the type of mortality information available, the ODE changes its type, which has implications about existence of general analytical solutions. In many epidemiologically relevant cases, an analytical solution does not exist, and numerical treatment has to be used instead.

An important application of the ODE is the derivation of age-specific incidence rates from the age distribution of the prevalence. This article shows that this question can be interpreted as an ill-posed inverse problem. The proof of the ill-posedness shows that an additive high-frequency distortion (*ε*  sin(*n*·)) of the prevalence may lead to an unbounded inaccuracy in the derived incidence. However, high-frequency distortions might be unlikely in real chronic diseases. Hence, the consequences in practical epidemiology are presumably small. In addition, it has recently been shown that, for the task of estimating incidence rates from prevalence data, methods based on ODEs, like the one presented in this article, are superior compared to other methods [21].

An application has been given from the context of type 1 diabetes in young women. The solution of the inverse problem showed a peak of the age-specific incidence rate at the age of about 28 years. Data about the incidence of type 1 diabetes in age 20+ are extremely scarce. Thus, the presented findings may gain insights into the epidemiology of type 1 diabetes.

In the discussed ODE model, several assumptions have been made. The ODE is valid only if incidence and mortality rates are independent of calendar time. In demography and epidemiology, this assumption is usually called *time homogeneity*. Due to changes in medical progress, hygiene, nutrition, and lifestyle, mortality does undergo secular trends. Thus, it is appropriate to formulate Equation (9) in terms of a partial differential equation (PDE) [22]. However, the associated PDE can be reduced to an ODE again by the *method of characteristics* [6]. By this, the presented results of this article remain valid in the case when time homogeneity does not hold.

Moreover, in real diseases, mortality of the diseased persons depends on the duration of the disease. An example is diabetes, where the relative mortality over the diabetes duration is U-shaped [23]. In this case, the theory of differential equations still can be applied if the mortality rate of the diseased is modified slightly [5].

Furthermore, the ODE (9) holds true only if the considered population is closed. In a recent article, we described the necessary changes to deal with immigration or emigration [10]. Thus, the results described here show a possible path of generalization to populations with migration.

A last note refers to the term “*chronic disease*.” In this article, *chronic* means *irreversible*, i.e., there is no way back from the *diseased* state to the *nondiseased* state. However, most of the results presented here remain true, if there is remission back to the state *nondiseased*. Then, the fundamental ODE (9) has an additional term that depends on the remission rate [20, 22].

## Figures and Tables

**Table 1:** Coefficients of Gompertz mortality rates *m*_0_ and *m*_1_.

Mortality rate	*β*_0_	*β*_1_
*m*_0_	−10.7	0.1
*m*_1_	−10.0	0.1

**Table 2:** Types of the ODE (9) depending on the given mortality.

Given mortality	Right-hand side of Equation (9)	Type of the ODE
*m* and *m*_0_	(1 − *p*){*i* − (*m* − *m*_0_)}	Linear
*m* and *m*_1_	(1 − *p*){*i* − *p*((*m*_1_ − *m*)/(1 − *p*))}	Linear
*m*_0_ and *m*_1_	(1 − *p*){*i* − *p*(*m*_1_ − *m*_0_)}	Riccati
*m*_0_ and *R*	(1 − *p*){*i* − *pm*_0_(*R* − 1)}	Riccati
*m*_1_ and *R*	(1 − *p*){*i* − *pm*_1_((*R* − 1)/*R*)}	Riccati
*m* and *R*	(1 − *p*){*i* − *m*((*p*(*R* − 1))/(*p*(*R* − 1)+1))}	Abelian

## Data Availability

Data presented in the example application have been obtained from the Deutsches Institut für Medizinische Dokumentation und Information (DIMDI). As a result of Germany's strict regulations on data protection, data are only available in an anonymous and aggregated form (§5 Data Transparency Regulation). Eligible research institutes according to §303e Section 1 German Code of Social Law, Book V, can obtain the aggregated data from the DIMDI after application and positive approval. The author and the affiliated institutes are not allowed to provide data access (§8 of Terms of Use).
